# Prony Analysis of Left Ventricle Pressure and Volume^✰^

**DOI:** 10.1016/j.medengphy.2023.103987

**Published:** 2023-05-02

**Authors:** Vinay P. Jani, Alexander T. Williams, Vivek P. Jani, Amy G. Tsai, Marcos Intaglietta, Pedro Cabrales

**Affiliations:** a Department of Bioengineering, University of California, San Diego, La Jolla, CA, 92093-0412, United States of America; b Division of Cardiology, Department of Medicine, Johns Hopkins University School of Medicine, Baltimore, MD, 21205, United States of America

**Keywords:** Prony analysis, Pressure-volume measurements, Hemorrhagic shock, Transfer function, Cardiovascular hemodynamics

## Abstract

Direct measurement of cardiac pressure-volume (PV) relationships is the gold standard for assessment of ventricular hemodynamics, but few innovations have been made to “multi-beat” PV analysis beyond traditional signal processing. The Prony method solves the signal recovery problem with a series of dampened exponentials or sinusoids. It achieves this by extracting the amplitude, frequency, dampening, and phase of each component. Since its inception, application of the Prony method to biologic and medical signal has demonstrated a relative degree of success, as a series of dampened complex sinusoids easily generalizes to multifaceted physiological processes. In cardiovascular physiology, the Prony analysis has been used to determine fatal arrythmia from electrocardiogram signals. However, application of the Prony method to simple left ventricular function based on pressure and volume analysis is absent. We have developed a new pipeline for analysis of pressure volume signals recorded from the left ventricle. We propose fitting pressure-volume data from cardiac catheterization to the Prony method for pole extraction and quantification of the transfer function. We implemented the Prony algorithm using open-source Python packages and analyzed the pressure and volume signals before and after severe hemorrhagic shock, and after resuscitation with stored blood. Each animal (*n* = 6 per group) underwent a 50% hemorrhage to induce hypovolemic shock, which was maintained for 30 min, and resuscitated with 3-week-old stored RBCs until 90% baseline blood pressure was achieved. Pressure-volume catheterization data used for Prony analysis were 1 s in length, sampled at 1000 Hz, and acquired at the time of hypovolemic shock, 15 and 30 min after induction of hypovolemic shock, and 10, 30, and 60 min after volume resuscitation. We next assessed the complex poles from both pressure and volume waveforms. To quantify deviation from the unit circle, which represents deviation from a Fourier series, we counted the number of poles at least 0.2 radial units away from it. We found a significant decrease in the number of poles after shock (*p* = 0.0072 vs. baseline) and after resuscitation (*p* = 0.0091 vs. baseline). No differences were observed in this metric pre and post volume resuscitation (*p* = 0.2956). We next found a composite transfer function using the Prony fits between the pressure and volume waveforms and found differences in both the magnitude and phase Bode plots at baseline, during shock, and after resuscitation. In summary, our implementation of the Prony analysis shows meaningful physiologic differences after shock and resuscitation and allows for future applications to broader physiological and pathophysiological conditions.

## 1. Introduction

Left ventricular pressure-volume (PV) loop analysis is the gold standard assessment of left ventricular hemodynamics and cardiac function, allowing for comprehensive assessment of systolic and diastolic function, changes in cardiac function in disease, and testing of ventricular support devices. Analysis of PV loops can be classified as “multi-beat,” which utilizes multiple beats throughout the cardiac cycle, or “single-beat,” which allows for estimation of systolic and diastolic function using a single cardiac cycle. However, analysis methods of these curves have been limited to traditional signal processing approaches in the time domain, with limited frequency domain applications. Prony’s method, or Prony’s analysis, is a method of digital signal processing that decomposes a given evenly sampled discrete signal into a series of dampened sinusoids [1,2]. The Prony method extracts the amplitude, frequency, dampening, and phase of each component by mathematically fitting a signal to the series,

(1) $$y\left[t_{k}\right]=\Sigma_{i=0}^{N-1}A_{i}e^{\sigma_{i}t_{k}}\cos\left(2\pi f_{i}t_{k}+\phi_{i}\right)$$

where $A_{i}$ is the amplitude of component $i,\;\sigma_{i}$ is the damping coefficient of component $i,\;\phi_{i}$ is the phase of component $i,\;f_{i}$ is the frequency of component i, and N is the number of components. Advances in computational methods and signal processing have allowed for expansion of Prony’s method for more complex signal processing tasks, including analysis of biological signals [3].

Prony’s method has several advantages compared to other computational methods despite its complexity. The Prony method shows reasonable predictive power. From a small segment of a signal, Prony’s methods computes its poles by the least squares methods, which completely describe its time dynamics [3]. The poles provided in the frequency domain can then be used to predicts its future time course. Compared to modern machine learning based methods, such an approach does not require large training data sets, highlighting yet another distinct advantage. In addition, Prony’s method computes an analytic function for any signal. Given that a series of dampened sinusoids has an analytic expression for both its Fourier transform and Laplace transform, the Prony method allows for an analytic expression for transfer functions.

For biologic and physiological signal processing, Prony’s method has been leveraged for the processing and understanding of complex biological behaviors and for several clinical applications. Prony’s method has been applied to multifocal visual-evoked potential for the diagnosis of multiple sclerosis [3] and analysis of electrocardiogram potentials for auto-discrimination of ventricular tachycardia, ventricular fibrillation, and supraventricular tachycardia [4]. For the latter application, the use of Prony’s method in conjunction with autoregressive modeling was superior to simple fast Fourier transform (FFT)-based methods and provided higher frequency resolution [4]. In two-dimensional signal processing and imaging, Prony’s method has been applied to magnetic resonance images for detection of abnormal brain tissue and image denoising [5]. Despite many medical applications, the Prony’s method has not been applied to left ventricle pressure and volume measurements [6,7].

In a previous study, our group used left ventricular pressure volume measurements to examine hemodynamics after hemorrhagic shock and more importantly in response to volume resuscitation with stored red blood cells (RBCs) [8], consistent with current massive transfusion protocols [9,10]. We demonstrated using left ventricular pressure volume measurements that resuscitation with stored RBCs impaired indices of left ventricular function resulting in a compensatory reduction in systemic vascular resistance, and an impairment in the minimum rate of ventricular pressure increase, suggestive of mild left ventricular diastolic dysfunction compared to alternative storage protocols [8]. In this study, we sought to utilize historical left ventricular pressure volume measurements, consisting of raw signals from multiple cardiac cycles, and analyze them using the Prony method. This approach has several advantages, as the dominant frequency components with the pressure-volume loop largely depend on heart rate. Moreover, higher frequency components related to vascular capacitance are well described. We additionally sought to design a methodology for analysis of poles from the Prony analysis as well as the mathematical theory for a transfer function for a system in which the Prony method is applied to both the input and output of said system. We utilized this methodology to determine a cardiac Prony transfer function to mathematically quantify hemodynamics in hypovolemic shock and after volume resuscitation, which are well described. We hope that use of left ventricular pressure volume measurements with the Prony’s method as well as the new mathematical theory developed will provide necessary foundations for future applications to more sophisticated biological processes.

## 2. Methods

### 2.1. Formulation of the Prony series

The Prony method fits an evenly sampled discrete signal into a series of dampened sinusoids of the form $y\left[t_{k}\right]=\Sigma_{i=0}^{N-1}A_{i}e^{\sigma_{i}t_{k}}cos\left(2\pi f_{i}t_{k}+\phi_{i}\right)$, where $A_{i}$ is the amplitude of component $i,\;\sigma_{i}$ is the damping coefficient of component $i,\;\phi_{i}$ is the phase of component $i,\;f_{i}$ is the frequency of component i, and N is the number of components. Utilizing Euler’s formula, the sinusoidal component $cos\left(2\pi f_{i}t_{k}+\phi_{i}\right)$ can be rewritten as follows,

(2) $$cos\left(2\pi f_{i}t_{k}+\phi_{i}\right)=\frac{1}{2}\left(e^{j\left(2\pi f_{i}t_{k}+\phi_{i}\right)}+e^{-j\left(2\pi f_{i}t_{k}+\phi_{i}\right)}\right)=\frac{1}{2}\left(e^{2\pi f_{i}t_{k}}e^{j\phi_{i}}+e^{-2\pi jf_{i}t_{k}}e^{-j\phi_{i}}\right)$$

Substituting Eq. (2) back into the series expansion described Eq. (1) yields

(3) $$y\left[t_{k}\right]=\Sigma_{i=0}^{N-1}\frac{A_{i}}{2}e^{\sigma_{i}t_{k}}\left(e^{2\pi jf_{i}t_{k}}e^{j\phi_{i}}+e^{-2\pi jf_{i}t_{k}}e^{-j\phi_{i}}\right)$$

The Prony series $y\left[t_{k}\right]$ described by Eq. (3) can be rewritten under the assumption that the signals are “real”

(4) $$y\left[t_{k}\right]=\Sigma_{i=0}^{N-1}\frac{A_{i}}{2}e^{j\phi_{i}}e^{\left(\sigma_{i}+2\pi jf_{i}\right)t_{k}}+\Sigma_{i=0}^{N-1}\frac{A_{i}}{2}e^{-j\phi_{i}}e^{-\left(\sigma_{i}+2\pi jf_{i}\right)t_{k}}=\Sigma_{i=0}^{N-1}A_{i}e^{j\phi_{i}}e^{\left(\sigma_{i}+2\pi jf_{i}\right)t_{k}}$$

Defining $\mu_{i}=e^{\left(\sigma_{i}+2\pi jf_{i}\right)}$ and $C_{i}=A_{i}e^{j\phi_{i}}$, where $\mu_{i}$ are the “poles” of the Prony fit and $C_{i}$ are the fitted constants, the Prony series can be simplified to the following form,

(5) $$y\left[t_{k}\right]=\Sigma_{i=0}^{N-1}C_{i}\mu_{i}^{t_{k}}$$

One of the advantages of the formulation of the Prony series described by Eq. (5) is that it enables the poles and fitted constants to be calculated by linear models, drastically reducing computation time. The mathematical formulation will be described briefly below but is derived in detail elsewhere [3,11–14].

### 2.2. Linear prediction model

Any input signal $y\left[t_{k}\right]$ can be expressed as a linear combination of all components prior to it. Let N be the desired number of poles/components of the Prony Model. Let N be the desired number of poles for the Prony fit. Each term $y\left[t_{l}\right]$, where l=N,…,2N−1 can be expressed as a linear combination of terms $y\left[t_{m}\right]$, where m=l−N,l−N+1,…,l−1, or

(6) $$y\left[t_{l}\right]=\Sigma_{m=1-N}^{l-1}a_{l-1-m}y\left[t_{m}\right]$$

From this formulation, the maximum number of terms to perfectly fit a signal is *floor*(*M*/2), where M is the number of terms in the signal (i.e., the Prony frequency decomposition must also abide by Nyquist’s criteria). The objective of the LPM is to find the constants $a_{m}$, which define the characteristic polynomial of the signal. Each $a_{m}$ can be determined by the following linear system

(7) $$\left[\begin{array}{c} y\left[t_{N}\right]\\ y\left[t_{N+1}\right]\\ \vdots \\ y\left[t_{2N-1}\right] \end{array}\right]=\left[\begin{array}{cccc} y\left[t_{N-1}\right] & y\left[t_{N-2}\right] & \cdots & y\left[t_{0}\right]\\ y\left[t_{N}\right] & y\left[t_{N-1}\right] & \cdots & y\left[t_{1}\right]\\ \vdots & \vdots & \ddots & \vdots \\ y\left[t_{2N-2}\right] & y\left[t_{2N-3}\right] & \cdots y\left[t_{N-1}\right] & \end{array}\right]\left[\begin{array}{c} a_{0}\\ a_{1}\\ \vdots \\ a_{N-1} \end{array}\right]$$

Define

(8) $$[d]=\left[\begin{array}{c} y\left[t_{N}\right]\\ y\left[t_{N+1}\right]\\ \vdots \\ y\left[t_{2N-1}\right] \end{array}\right],\;[D]=\left[\begin{array}{cccc} y\left[t_{N-1}\right] & y\left[t_{N-2}\right] & \cdots & y\left[t_{0}\right]\\ y\left[t_{N}\right] & y\left[t_{N-1}\right] & \cdots & y\left[t_{1}\right]\\ \vdots & \vdots & \ddots & \vdots \\ y\left[t_{2N-2}\right] & y\left[t_{2N-3}\right] & \cdots y\left[t_{N-1}\right] & \end{array}\right],\;[a]\;=\left[\begin{array}{c} a_{0}\\ a_{1}\\ \vdots \\ a_{N-1} \end{array}\right]$$

The system in Eq. (7) can thus be rewritten as

(9) [d]=[D][a]

where [D] is the Hankel matrix and [a] is the linear prediction method. In the case when rank is equivalent to the row-rank (i.e., the matrix is square), the Hankel matrix is invertible. As the signal analyzed is real, the Hankel matrix is left invertible, thus allowing the linear prediction terms to be determined as $\left[a]={\left[D\right]}^{-1}[d\right]$ [13–15].

### 2.3. Determination of the poles

The poles $\mu_{i}$ are determined from the linear prediction coefficients [a], described elsewhere [12]. Briefly, [a] can be used to define the coefficients of the characteristics polynomial of the system, whose roots are indeed the poles. Defining the polynomial $P\left(x^{n}\right)$ with coefficients $a_{i}$ and roots $\mu_{i}$, we have

(10) $$P\left(x^{n}\right)=x^{N-1}-a_{0}x^{N-2}-a_{1}x^{N-3}-\ldots -a_{N-1}=\left(x-\mu_{0}\right)\left(x-\mu_{1}\right)\ldots \left(x-\mu_{N-1}\right)$$

The roots of this polynomial were numerically determined by calculation of the companion matrix [16].

### 2.4. Determination of fit coefficients

To determine the fit coefficients, we fit a linear combination of the poles to the original signal with weights $C_{i}$. In an M component signal with N Prony terms, where N≤floor(M/2), each term $y\left[t_{k}\right]$ can be expressed as a function of the poles to calculate the Prony series. In this way, the following linear system can be used to calculate the fitted parameters $C_{i}$

(11) $$\left[\begin{array}{c} y\left[t_{0}\right]\\ y\left[t_{1}\right]\\ \vdots \\ y\left[t_{M-1}\right] \end{array}\right]=\left[\begin{array}{cccc} \mu_{0}^{t_{0}} & \mu_{1}^{t_{0}} & \cdots & \mu_{N-1}^{t_{0}}\\ \mu_{0}^{t_{1}} & \mu_{1}^{t_{1}} & \cdots & \mu_{N-1}^{t_{1}}\\ \vdots & \vdots & \ddots & \vdots \\ \mu_{0}^{t_{M-1}} & \mu_{1}^{t_{M-1}} & \cdots & \mu_{N-1}^{t_{M-1}} \end{array}\right]\left[\begin{array}{c} C_{0}\\ C_{1}\\ \vdots \\ C_{N-1} \end{array}\right]$$

Defining

(12) $$\left[Y\right]=\left[\begin{array}{c} y\left[t_{0}\right]\\ y\left[t_{1}\right]\\ \vdots \\ y\left[t_{M-1}\right] \end{array}\right],[U]=[V{]}^{T}=\left[\begin{array}{cccc} \mu_{0}^{t_{0}} & \mu_{1}^{t_{0}} & \cdots & \mu_{N-1}^{t_{0}}\\ \mu_{0}^{t_{1}} & \mu_{1}^{t_{1}} & \cdots & \mu_{N-1}^{t_{1}}\\ \vdots & \vdots & \ddots & \vdots \\ \mu_{0}^{t_{M-1}} & \mu_{1}^{t_{M-1}} & \cdots & \mu_{N-1}^{t_{M-1}} \end{array}\right],[C]\;=\left[\begin{array}{c} C_{0}\\ C_{1}\\ \vdots \\ C_{N-1} \end{array}\right]$$

the system is written as $\left[Y]={\left[V\right]}^{T}[C\right]$, where [V] is the (N×M) vandermond matrix determined by the characteristic polynomial of the system [12,17,18]. The fit parameters $C_{i}$ are numerically determined by taking the Moore-Penrose pseudoinverse of the transpose of the vandermond matrix of the characteristic polynomial $\left[C]={\left([V{]}^{T}\right)}^{-1}[Y\right]$.

Both $\mu_{i}$ and $C_{i}$ are complex components that define the Prony Series. From these complex terms, it is possible to determine the amplitude, damping, phase, and frequency for each component in the Prony Series. From the poles $\mu_{i}$, we have

(13) $$\mu_{i}=Re\left(\mu_{i}\right)+jIm\left(\mu_{i}\right)=e^{\left(\sigma_{i}+2\pi jf_{i}\right)}=e^{\sigma_{i}}e^{2\pi jf_{i}}$$

Thus, the poles $\mu_{i}$ can be rewritten as

(14) $$\mu_{i}=\mu_{i}e^{Ph\left(\mu_{i}\right)}=e^{\sigma_{i}}e^{2\pi f_{i}}$$

where $Ph\left(\mu_{i}\right)={tan}^{-1}\left(\frac{Im\left(\mu_{i}\right)}{Re\left(\mu_{i}\right)}\right)$ and $\mu_{i}$ is its magnitude. By inspection, we see

(15) $$\sigma_{i}=ln\left(\mu_{i}\right)\;f_{i}=\frac{1}{2\pi }Ph\left(\mu_{i}\right)$$

Using a similar approach, we have

(16) $$C_{i}=Re\left(C_{i}\right)+jIm\left(C_{i}\right)=A_{i}e^{j\phi_{i}}\cdot C_{i}=C_{i}e^{jPh\left(C_{i}\right)}=A_{i}e^{j\phi_{i}}$$

Thus, the amplitude and phase of the Prony series are

(17) $$\;A_{i}=C_{i}\;\phi_{i}=Ph\left(C_{i}\right)$$

### 2.5. Prony fit convergence

The Vandermonde matrix in Eq. (12) used to determine the fit coefficients $C_{i}$ is sensitive to the magnitude of the time step $t_{k}$. For high sampling rates, the Vandermonde matrix can have low rank order [19, 20]. To compensate for this limitation, the time $t_{k}$ is scaled by the sampling rate and some factor d. The Prony series in our implementation therefore takes the form

(18) $$y\left[t_{k}\right]=\Sigma_{i=1}^{N-1}C_{i}\mu_{i}^{t_{k}\times SR/d}$$

where SR is the sampling rate and d is arbitrary. d is optimized by maximizing the coefficient of determination $R^{2}$ between the Prony fit and the raw signal across all d∈R by means of gradient ascent [21].

The Prony algorithm described was implemented using the numpy [22] and scipy [23] base packages in Python and will be made publicly available. A graphical user interface (GUI) implementing the Fourier series decomposition and the Prony fit (along with simple signal processing algorithms) was also constructed using the Python tkinter [24] library, which will also be made publicly available.

### 2.6. Animal experiments

The complete protocol for acquisition of the rodent pressure and volume signals used for Prony Analysis are described in detail elsewhere [8]. Briefly, male Sprague-Dawley rats, which weighed about 200–250 g (Harlan Laboratories, Indianapolis, Ind), were hemorrhaged for a span of 30 min via withdrawing a total of 50% of the animal’s blood volume (estimated by taking 7% of the animal’s body weight) and resuscitated after 30 min with blood that was stored for 3 weeks with conventional storage practices followed including storage at 4 ° Celsius. The hemorrhagic shock and resuscitation protocols are described in detail elsewhere [8]. For all animals in this study, the NIH Guide for the Care and Use of Laboratory Animals was followed. The study protocol was approved by the local animal care committee. For the duration of the experiment, rats were anesthetized with isoflurane in compressed room air (Drägerwerk AG, Lübeck, Germany), and were concurrently on a heating pad for maintenance of core temperature (37 °Celsius). Pressure and volume signals used for further signal processing were collected via a 2F pressure volume (PV) conductance catheter (SPR-858; Millar Instruments, Houston, Tex), which was inserted into the left ventricle (LV) via the close chested method with signal continuously received (MPVS300; Millar Instruments, and PowerLab 8/30, ADInstruments, Colorado Springs, Colo) [25]. The closed chest method involves isolation of the right carotid artery, and the PV catheter advancing through the aorta.

### 2.7. Statistical analysis

All data are represented as mean±SD. The raw signal and each Prony fit were compared with the coefficient of determination R^2^ determined using a custom algorithm in Python, as was the Fourier series decomposition. Frequency spectra for the signal were calculated using a fast Fourier transform in Python. Quantification of complex pole analysis between all groups (baseline, shock, and resuscitation) is discussed in detail below. Results from this analysis were compared using one-way repeated measures ANOVA and mixed effect models with Holm-Sidak’s test for post-hoc comparison when appropriate. Bode plots were analytically deriving expressions for the transfer function for the pressure-volume relationship in the Laplace domain and numerically calculating the magnitude and phase for all groups between 1 and 100 Hz with resolution of 1 Hz. Composite Bode plots were determined by simple averaging. All statistical analysis performed in GraphPad Prism 9.1 (GraphPad Software, San Diego, CA) or Python base packages (numpy and scipy). Changes were considered significant if p values were < 0.05.

## 3. Results

We performed Prony analysis utilizing left ventricular pressure volume measurements given its cyclic nature and well described time dynamics before and after induction of hypovolemic shock and followed by volume resuscitation with stored RBCs. A total of six (*n* = 6) animals per group were included for analysis. Each animal underwent a 50% hemorrhage to induce hypovolemic shock, which was maintained for 30 min, and resuscitated with 3-week-old stored RBCs until 90% baseline MAP was achieved. Pressure-volume catheterization data used for Prony analysis were 1 s in length, sampled at 1000 Hz, and acquired at the time of hypovolemic shock, 15 and 30 min after induction of hypovolemic shock, and 10, 30, and 60 min after volume resuscitation. All animals survived the protocol, and all groups were similar at baseline as determined by Grubb’s method. All animals had similar MAP at baseline and after hemorrhage. Detailed hemodynamics are provided elsewhere [8].

### 3.1. Application of the Prony method for pressure-volume signal processing

**Fig. 1.**
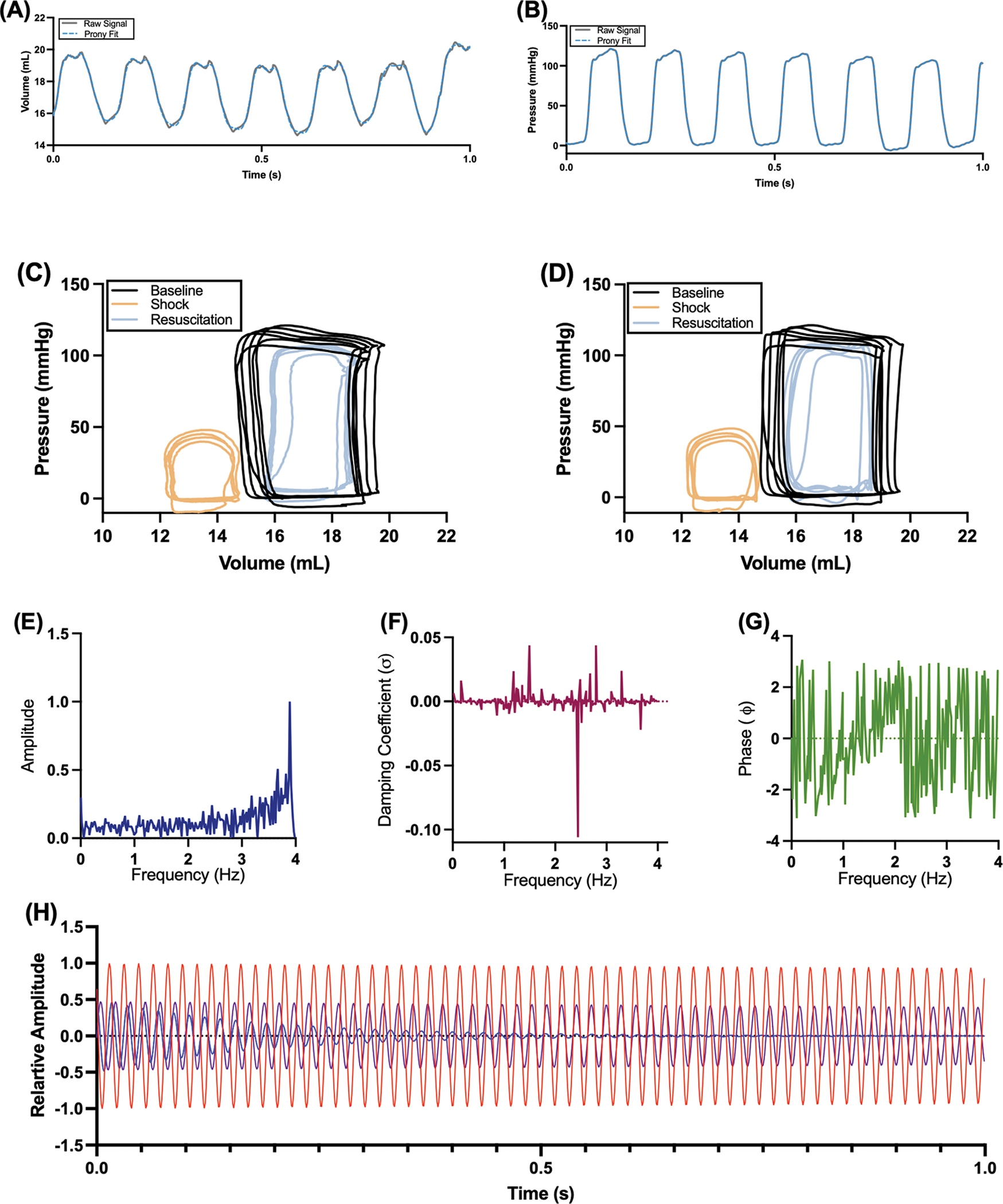
Application of Prony’s method to pressure-volume measurement catheterization data. (A) Prony fit applied to the volume vs. time waveform (R^2^ > 0.99). (B) Prony fit applied to the pressure vs. time waveform (R^2^ > 0.99). (C) Representative pressure volume measurements s at baseline (black), shock (beige), and resuscitation (blue). (D) Pressure volume measurements s derived from the Prony fit at baseline (black), shock (beige), and resuscitation (blue). These data demonstrate that we successfully implemented the Prony algorithm for our signals. Representative (E) Amplitude, (F) Dampening coefficient, and (G) Phase spectra for a single pressure waveform, demonstrating the potential outputs of the Prony algorithm. (H) Prony decomposition of the top 4 most significant dampened sinusoidal components for a single pressure waveform at baseline.

To each pressure-volume catheterization signal, the Prony method was applied and fit coefficients for the corresponding series of dampened sinusoids were determined. The number of Prony terms was a hyperparameter, optimized by maximizing $R^{2}$ between the Prony fit and the raw signal, and was between 340 and 350 terms for all signal assessed. For all fits, $R^{2}>0.99$. A representative output of the Prony method is shown in Fig. 1. As demonstrated, we fit the pressure waveform (Fig. 1A), volume waveform (Fig. 1B), and replicated a pressure volume (PV) measurement using the Prony fits (Fig. 1D), demonstrating the relative success of our implementation. Given that the Prony method fits the signal to a series of dampened sinusoids $f[t]=\sum_{i}A_{i}e^{\sigma_{i}t}cos\left(2\pi f_{i}t+\right.\phi_{i})$, then in addition to the frequency spectrum A[f] and the phase spectrum ϕ[f], the Prony method provides a frequency spectrum for the dampening coefficient, σ[f]. Importantly, this spectral feature is absent within a Fourier analysis. For a representative pressure signal, these spectra are shown in Fig. 1E–G. A representative Prony decomposition for the same pressure signal for the four most prominent dampened sinusoids, as determined by their relative amplitudes is shown in Fig. 1H. These data demonstrate that for a given signal, rather than fitting a dampened sinusoid with frequency identical to the signal, in this case the heart rate, the Prony method opts to fit higher frequency signals out of phase with dampening.

### 3.2. Comparison between the Fourier transform and the Prony method

**Fig. 2.**
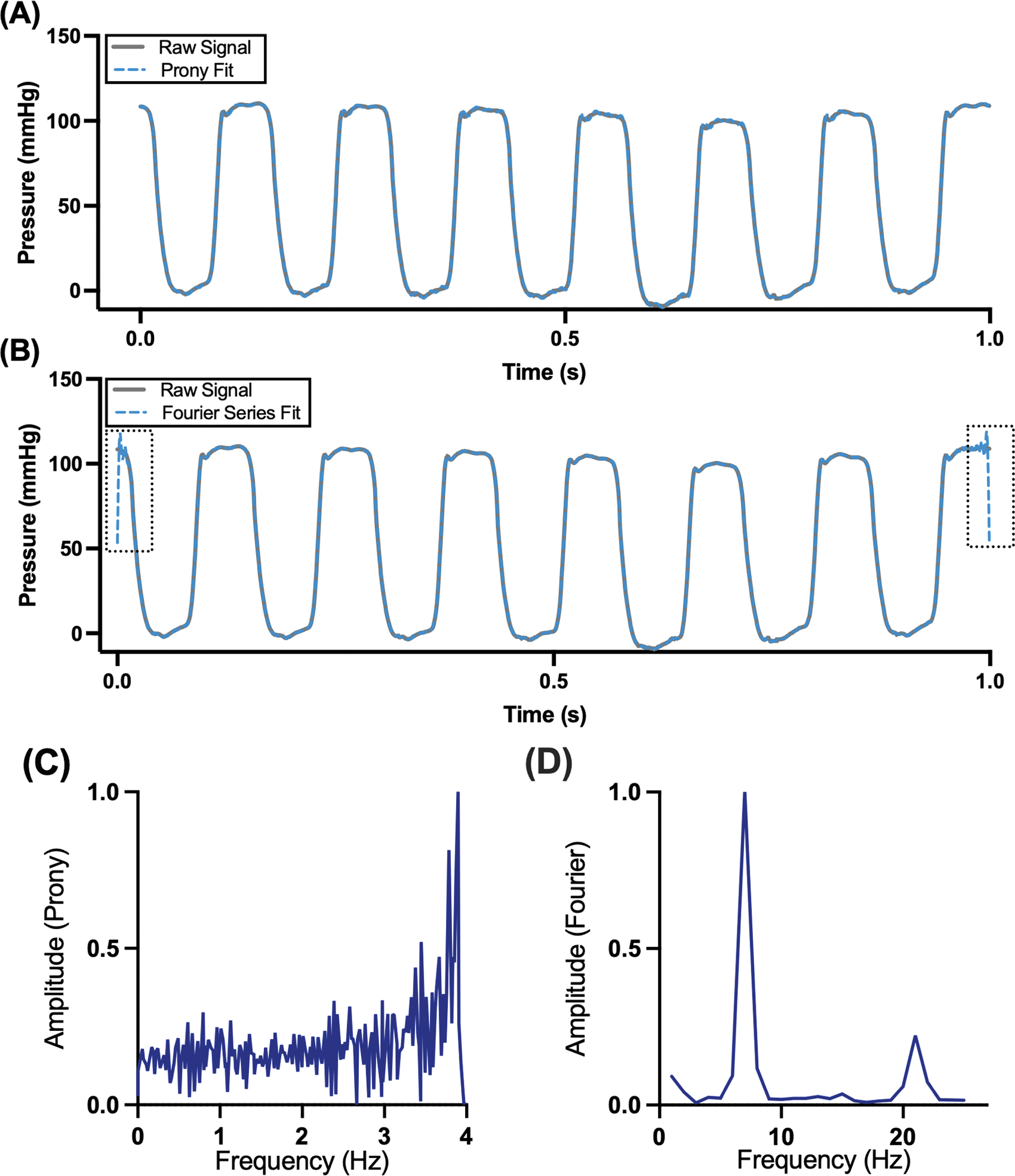
Comparison between the Prony fit and the Fourier series decomposition. We implemented both a (A) Prony fit (R^2^ > 0.99) and (B) Fourier series decomposition (R^2^ > 0.99) for a single pressure waveform at baseline. Compared to the Prony fit, the Fourier series decomposition demonstrated significant edge effects (boxed). Frequency spectra for the (C) Prony decomposition and (D) Fourier series decomposition were also calculated to highlight key differences.

We next sought to distinguish the outputs from the Prony method to those from traditional Fourier transform and Fourier series decomposition-based signal processing methods. These differences are highlighted in Fig. 2. Both the Fourier series fit and the Prony fit closely aligned with the raw signal ($R^{2}>0.99$). One advantage to the Prony fit is that edge effects are minimal contrasted with the Fourier series fit (Fig. 2B). Next, we compared the frequency power spectra for both the Prony and Fourier fit, presented in Fig. 2C and D, respectively. Here, we observed that the power spectral density from a fast Fourier transform represents the dominant frequencies that compose the figure. These frequencies are less than half of the normal rodent heart rate and are therefore likely not influenced by it. Specifically, we observe a peak at approximately 7 Hz, corresponding with the heart rate of the animals in our experiment (~420 bpm) as well as a higher frequency harmonic. The frequencies identified from the Prony power spectral density do not represent physical frequencies (Fig. 2C).

### 3.3. Complex pole analysis in the Prony domain

**Fig. 3.**
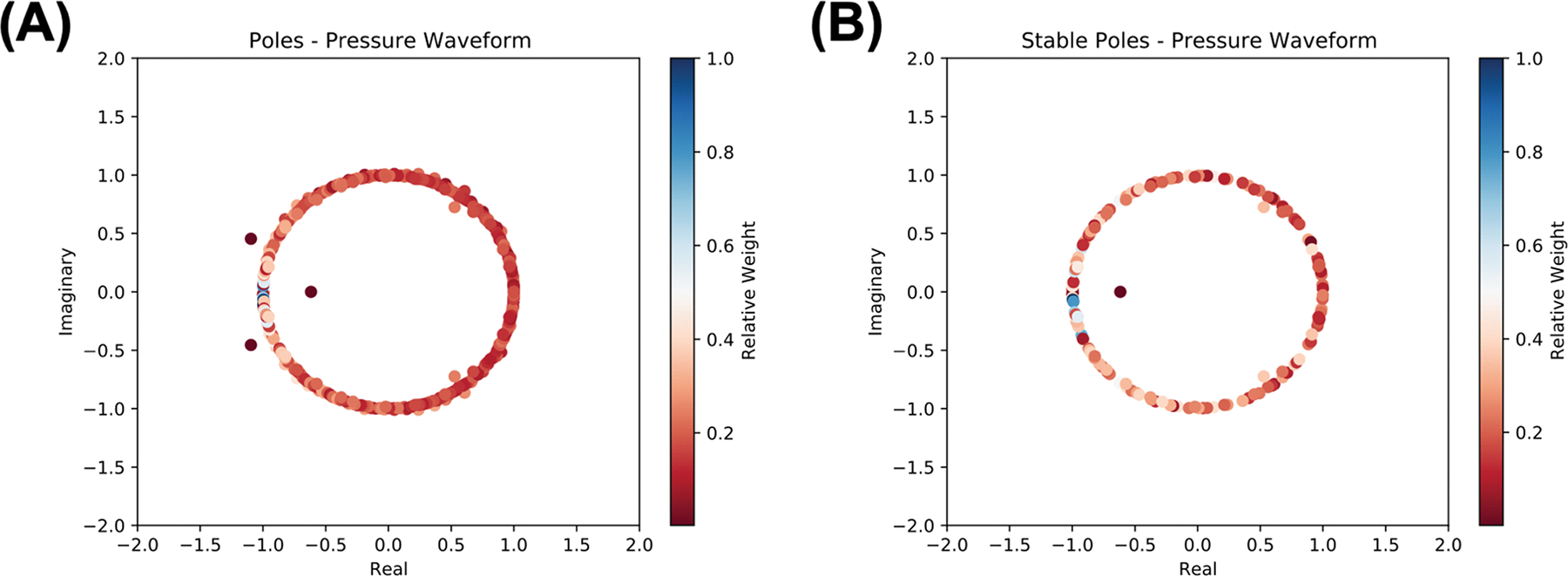
Representative pole diagrams for a single pressure waveform at baseline in the real imaginary plane. A Prony fit containing (A) all poles and (B) only stable poles were fit and shown in the real-imaginary plane. Each point represents a pole of the pressure waveform Prony fit, and the color corresponds with relative weight of the pole, quantified as the magnitude of the fit coefficient C. Relative weights were normalized to the fit coefficient with the highest magnitude. A color of red indicates low relative importance, while a color of blue indicates more importance in this representation.

We next analyzed the complex poles obtained from the Prony fit. Such analysis will allow for identification of the number of stable and unstable dampened sinusoids that make up the signal. Recall that in the method for Prony analysis we implemented, signals were fit to dampened sinusoids of the form $f[t]=\sum_{i}C_{i}\mu_{i}^{t}$, where $C_{i}$ and $\mu_{i}$ are complex. Here, we denote $\mu_{i}$ as the poles. Each pole was plotted in the real-imaginary plane and colored with relative importance quantified as $\left\|C_{i}\|/\underset{i}{max}\|C_{i}\right\|$. A representative pole diagram is shown in Fig. 3A. Poles corresponding to $\sigma_{i}>0$ are unstable while those corresponding to $\sigma_{i}\leq 0$ are stable. Graphically in the real-imaginary plane, as $\sigma_{i}=ln\left\|\mu_{i}\right\|$, poles on or inside the unit circle would be considered stable, while those outside the unit circle, unstable. In the specific case when $\sigma_{i}=0$, then $f[t]=\sum_{i}C_{i}\mu_{i}^{t}=\sum_{i}A_{i}cos\left(2\pi f_{i}t+\phi_{i}\right)$. Poles on the unit circle correspond to cosine waves without dampening and generating poles on the unit circle would indeed result in a Fourier series decomposition.

We used complex pole analysis to identify only stable poles and re-fit a series of dampened sinusoids including only those stable poles. An alternative Prony fit utilizing only stable poles allows for the generation of a signal that will not diverge, if the signal is used for forward-time signal extrapolation, one known, potential application of the Prony method [3]. For all signals 40–60 poles were unstable and removed, resulting in Prony fits with 275–315 terms. $R^{2}>0.99$ for all Prony fits with only stable poles suggesting that such an analysis successfully recapitulated the signal. We plotted pole diagrams from these new Prony fits with only stable poles, which demonstrated that the weights (magnitude of C) on average increased and became more disperse after eliminating unstable poles. These results suggest that unstable poles encode large amounts of information, which when eliminated, significantly alter the dampened sinusoidal decomposition. A representative diagram with this analysis is shown in Fig. 3B.

### 3.4. Complex pole analysis pre and post volume resuscitation in a rodent model of hypovolemic shock

We quantified important features from pole diagrams in the real imaginary plane to examine correlations with either hypovolemic shock or resuscitation. Specifically, we designed two methods to quantify (i) the location of those poles deemed most significant based on the amplitude of the constant C from the Prony method and (ii) pole stability. Each pole has a relative importance quantified as $\left\|C_{i}\right\|/{max}_{i}\|C_{i}\|$, which we will denote $\alpha_{i}$. The location of a pole in the real-imaginary plane can be quantified by a single angle $\theta_{i}={tan}^{-1}\left(Im\left(\mu_{i}\right)/Re\left(\mu_{i}\right)\right)$. We quantify the location, or mean angle, of the most significant poles in the real imaginary axis as the weighted average of all $\theta_{i}$ with weights $\alpha_{i}$, or

(19) $$\bar{\theta }=\frac{\sum_{i}\alpha_{i}\theta_{i}}{\sum_{i}\theta_{i}}$$

**Fig. 4.**
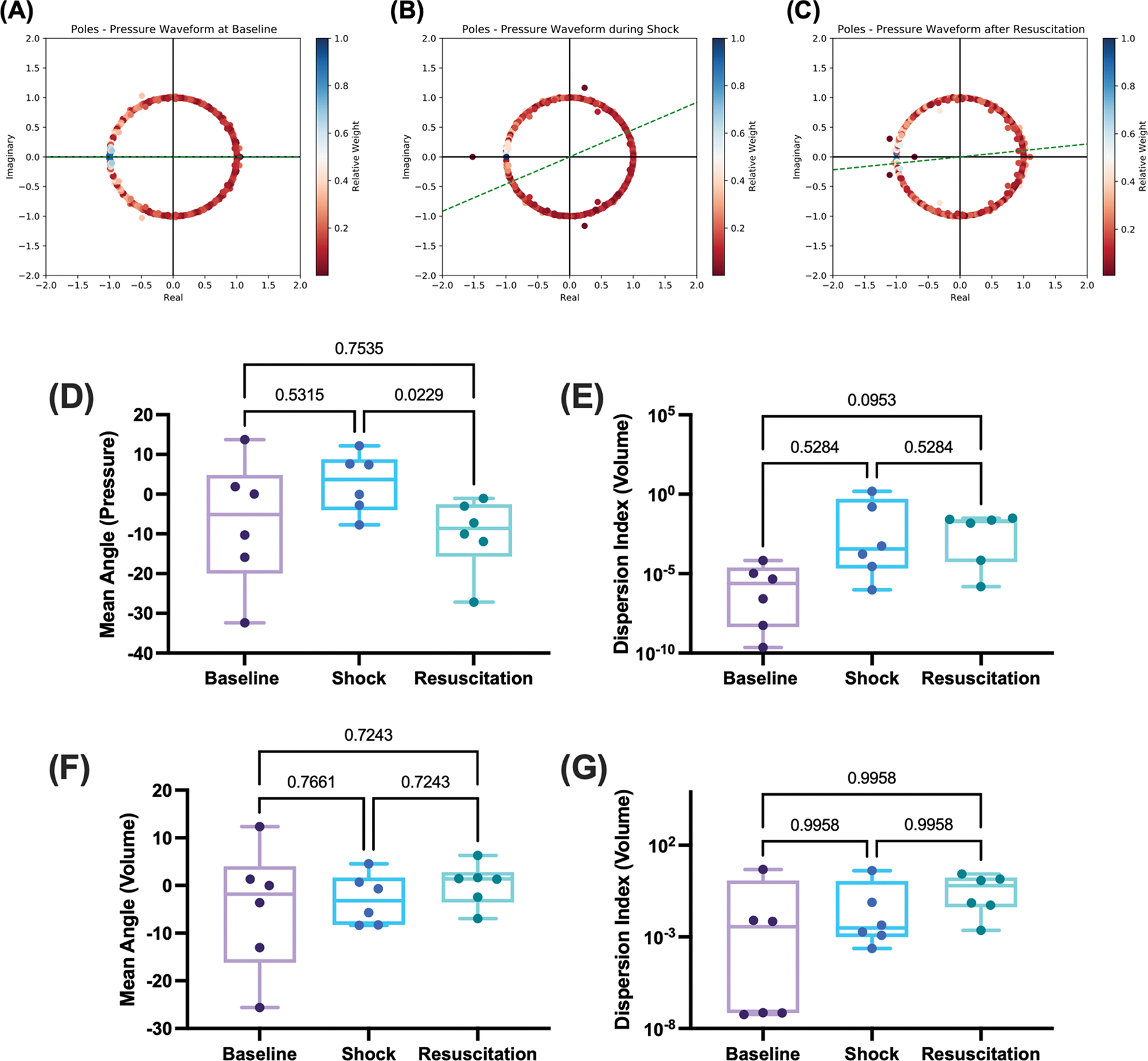
Complex pole analysis at baseline, shock, and resuscitation for both stable and unstable Prony poles. Representative pole diagrams in the real-imaginary planes (A) at baseline, (B) during hemorrhagic shock, and (C) after volume representation. Poles are plotted in the real-imaginary plane with relative importance of each pole assessed by the magnitude of the Prony fit coefficient. For each pole diagram, the line corresponding to the angle of orientation of the most significant poles is shown in green. Representative angles plotted for one case at baseline (mean angle 0°), during hypovolemic shock (mean angle 26°), and after volume resuscitation (mean angle 6°) for poles of the pressure waveform are shown. Summary data for the (D) mean angle and (E) dispersion for the pressure waveforms, and (F) mean angle and (G) dispersions for the volume waveform are shown. Results from this analysis were compared using one-way repeated measures ANOVA and mixed effect models with Holm-Sidak’s test for post-hoc comparison when appropriate.

We next quantified the “dispersion” of the poles simply as the inverse of the standard deviation of the weights $\left\|C_{i}\right\|$, or $1/\sigma_{i}$. A low dispersion indicates that all the poles were equally weighted in the Prony series decomposition, while a high dispersion indicates that a few poles predominate the decomposition, with the others providing little to no additional information. The location (mean angle) and dispersion were next quantified at baseline, during hypovolemic shock, and after volume resuscitation, considering all poles, stable and unstable within the Prony analysis. These results are summarized in Fig. 4, with representative angles plotted for one case at baseline (mean angle 0°), during hypovolemic shock (mean angle 26°), and after volume resuscitation (mean angle 6°) for poles of the pressure waveform. We found a significance decrease in the mean angle of the poles between the shock and resuscitation time points (*p* = 0.0229 from ANOVA). No other significant differences were observed for the dispersion index and mean angle for the volume waveform. We repeated this analysis only for the stable poles in the Prony domain and observed no differences in either index. These results along with detailed statistical analysis are summarized in Supplemental Figure 1.

**Fig. 5.**
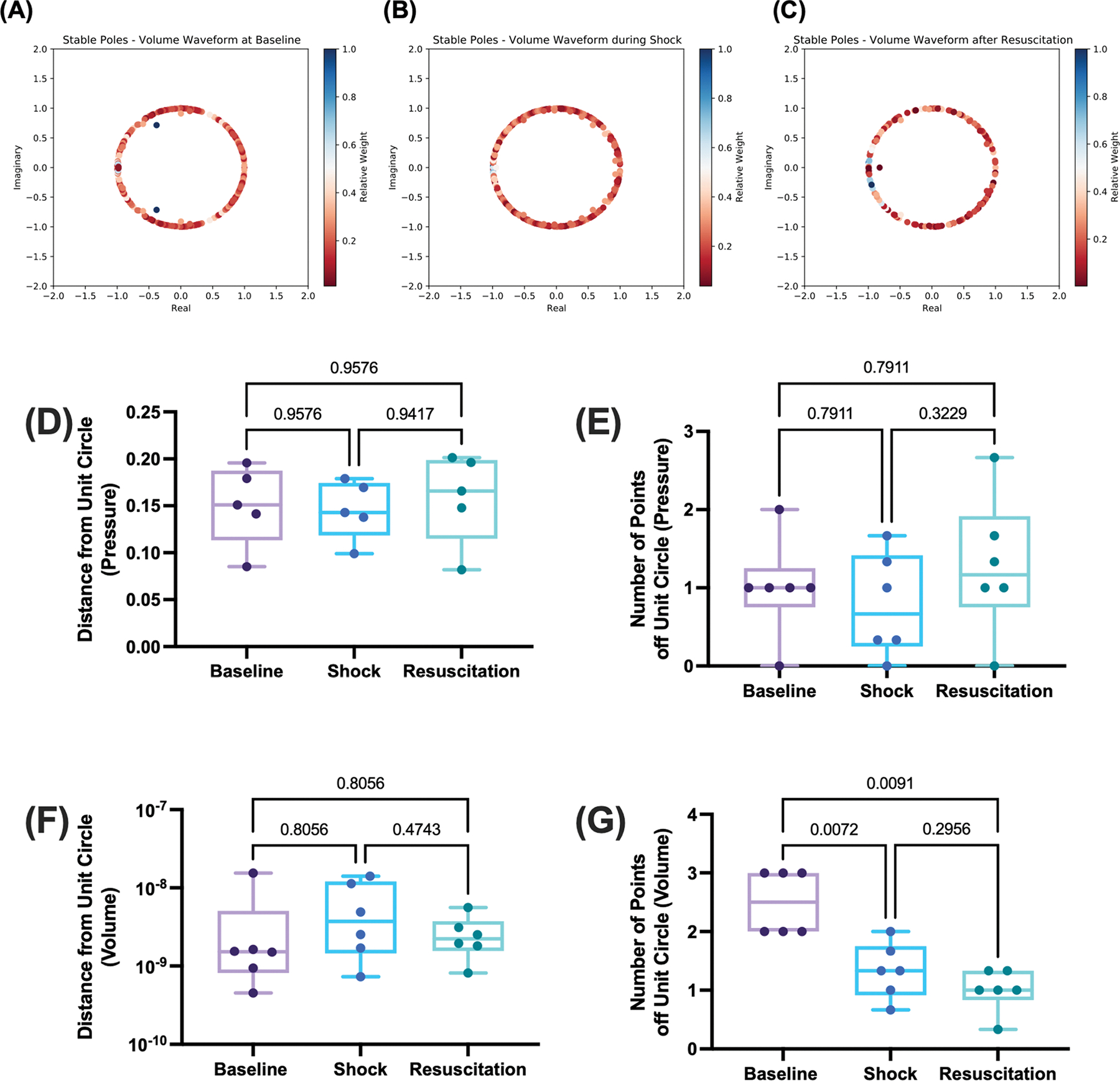
Complex pole analysis at baseline, shock, and resuscitation for only stable Prony poles. Representative pole diagrams in the real-imaginary planes (A) at baseline, (B) during hemorrhagic shock, and (C) after volume representation. Poles are plotted in the real-imaginary plane with relative importance of each pole assessed by the magnitude of the Prony fit coefficient. Summary data for the (D) mean distance for the top five further poles and (E) number of poles off the unit circle for the pressure waveforms, and (F) mean distance for the top five further poles and (G) number of poles off the unit circle volume waveform are shown. Results from this analysis were compared using one-way repeated measures ANOVA and mixed effect models with Holm-Sidak’s test for post-hoc comparison when appropriate.

We next quantified deviation from the unit circle in the real-imaginary axis as a proxy for significant dampening. We performed this analysis solely on stable poles ($\sigma_{i}\leq 0$) to allow for stratification in our analysis between stable and unstable poles. These results are summarized in Fig. 5. To quantify deviation, we utilized two metrics, namely the mean distance from the unit circle of the top 5 poles furthest away and the number of poles off the unit circle (defined as at least 0.2 units away in either direction). For stable pressure poles, we found no significant differences between all groups for either metric (*p* = 0.5498 for the distance metric and *p* = 0.4410 for the number of poles from the unit circle). Surprisingly, we found a significant difference in the number of stable poles off the unit circle for the volume waveform (*p* = 0.0014) and the number of poles after shock (*p* = 0.0072 vs. baseline) and after resuscitation (*p* = 0.0091 vs. baseline). No differences were observed in this metric pre and post volume resuscitation (*p* = 0.2956). Representative pole diagrams for volume waveforms demonstrating this phenomenon are shown in Fig. 5A–C and summary statistics are presented in Fig. 5D–G. We also repeated this analysis for all poles for both the pressure and volume waveforms, which revealed no differences. Detailed statistical analysis is presented in Supplemental Figure 2.

### 3.5. Investigation of pressure-volume relationships using the Prony method

**Fig. 6.**
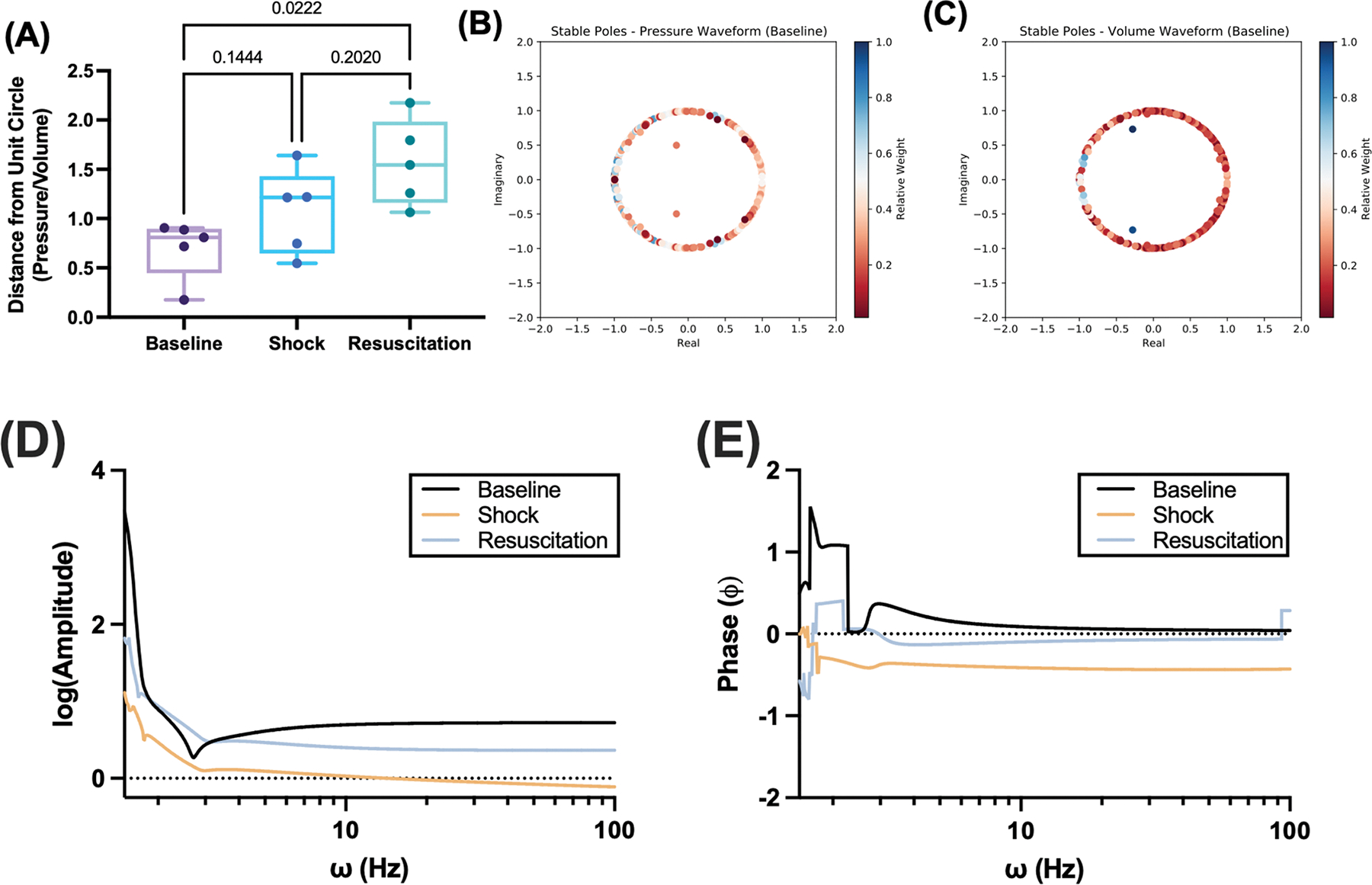
Pressure-volume relationships during hemorrhagic shock and after volume resuscitation. (A) The ratio of the distance from the unit circle for pressure and volume waveforms, with the top 5 most significant poles in each. Results from this analysis were compared using one-way repeated measures ANOVA and mixed effect models with Holm-Sidak’s test for post-hoc comparison when appropriate. Representative “pseudo-pole” diagrams for the (B) pressure and (C) volume waveforms. Composite (D) magnitude and (E) phase Bode plots numerically determined from analytic forms of the Laplace transform of the Prony series. Bode plots were plotted between 1 and 100 Hz and averaged for all animals at baseline (black), during shock (beige), and after resuscitation (blue).

The pressure and volume waveforms vary intrinsically with elastance during isovolumic contraction and ejection and passive mechanical properties during diastole. We quantified the ratio of the number of stable poles with significant dampening using the same metrics as in Fig. 5. These results are summarized in Fig. 6A. Briefly, we found a significant increase in this ratio after resuscitation (*p* = 0.0222 vs. baseline). A similar analysis was performed all the aforementioned metrics, shown in Supplemental Figure 3. Visual inspection of pole diagrams also reveals that for the same animal at the same time, the pressure and volume pole diagrams in the real-imaginary axis appear to be complementary (Fig. 6B, C).

As dampened sinusoids have analytically defined Laplace transforms, we determined a transfer function that describes the pressure-volume relationship for the heart at baseline, after hemorrhagic shock, and after volume resuscitation. Recall that the Prony method fits a given signal to the series $f[t]=\sum_{i}C_{i}\mu_{i}^{t}=\sum_{i}A_{i}e^{\sigma_{i}t}cos\left(2\pi f_{i}t+\phi_{i}\right)$. Let $P\left[t_{k}\right]=\sum_{i=0}^{N-1}A_{i}^{\left(P\right)}e^{\sigma_{i}^{\left(P\right)}t_{k}}cos(2\pi f_{i}^{\left(P\right)}t_{k}+\phi_{i}^{\left(P\right)})$ be the Prony fit for the pressure waveform and $V\left[t_{k}\right]=\sum_{i=0}^{N-1}A_{i}^{\left(V\right)}e^{\sigma_{i}^{\left(V\right)}t_{k}}cos(2\pi f_{i}^{\left(V\right)}t_{k}+\phi_{i}^{\left(V\right)})$ be the corresponding Prony fit for the volume waveform. For a dampened sinusoid, the Laplace transform can be analytically expressed as

(20) $$\bar{f}\left[s_{k}\right]=\sum_{i=0}^{N-1}A_{i}\left(\frac{cos\phi_{i}\left(s_{k}\;-\;\sigma_{i}\right)\;-\;2f_{i}\pi sin\sigma_{i}}{{\left(s_{k}\;-\;\sigma_{i}\right)}^{2}\;+\;4f_{i}^{2}\pi^{2}}\right)$$

A pressure-volume transfer function can therefore be developed:

(21) $$\bar{Z}\left(s_{k}\right)=\frac{\sum_{i=0}^{N-1}A_{i}^{\left(P\right)}\left(\frac{cos\phi_{i}^{\left(P\right)}\left(s_{k}-\sigma_{i}^{\left(P\right)}\right)-2f_{i}^{\left(P\right)}\pi sin\phi_{i}^{\left(P\right)}}{{\left(s_{k}-\sigma_{i}^{\left(P\right)}\right)}^{2}\;+4f_{i}^{(P)2}\pi^{2}}\right)}{\sum_{i=0}^{N-1}A_{i}^{\left(V\right)}\left(\frac{cos\phi_{i}^{\left(V\right)}\left(s_{k}-\sigma_{i}^{\left(V\right)}\right)-2f_{i}^{\left(V\right)}\pi sin\phi_{i}^{\left(V\right)}}{{\left(s_{k}-\sigma_{i}^{\left(V\right)}\right)}^{2}\;+4f_{i}^{(V)2}\pi^{2}}\right)}$$

At any given frequency, the transfer function is proportional to either the time varying elastance or passive ventricular properties depending on the phase of the cardiac cycle.

The zeros and poles of this transfer function may reflect load independent indices, (e.g., cardiac power, stroke work, contractility, etc.). To test this hypothesis, we sought to look at the relationship between the poles of the transfer function and changes in contractility in response to hypovolemic shock. The transfer function in the Laplace domain can alternatively take the form,

(22) $$\bar{Z}\left(s_{k}\right)=\frac{\sum_{i}\frac{C_{i}^{\left(P\right)}}{s_{k}-\mu_{i}^{\left(P\right)}}}{\sum_{i}\frac{C_{i}^{\left(V\right)}}{s_{k}-\mu_{i}^{\left(V\right)}}}=\frac{\left(\sum_{i}C_{i}^{\left(P\right)}\prod_{j\neq i}s_{k}\;-\;\mu_{j}^{\left(P\right)}\right)\left(\prod_{i}s_{k}\;-\;\mu_{i}^{\left(V\right)}\right)}{\left(\sum_{i}C_{i}^{\left(V\right)}\prod_{j\neq i}s_{k}\;-\;\mu_{j}^{\left(V\right)}\right)\left(\prod_{i}s_{k}\;-\;\mu_{i}^{\left(P\right)}\right)}$$

The transfer function is in a “pseudo pole-zero” form, with poles defined by $\left(\prod_{i}s_{k}-\mu_{i}^{\left(P\right)}\right)=0$ and $\left(\sum_{i}C_{i}^{\left(V\right)}\prod_{j\neq i}s_{k}-\mu_{j}^{\left(V\right)}\right)=0$ and zeros defined by $\left(\sum_{i}C_{i}^{\left(P\right)}\prod_{j\neq i}s_{k}-\mu_{j}^{\left(P\right)}\right)=0$ and $\left(\prod_{i}s_{k}-\mu_{i}^{\left(V\right)}\right)=0$. The poles of the pressure waveform are poles of the transfer function and that the poles of the volume waveform are the zeros of the transfer function. The poles of the volume waveform encode information of the poles of the transfer function, but in a complex non-linear way that cannot be analytically simplified and likewise for the poles of the pressure waveform and the zeros of the transfer function. We therefore denote the pressure and volume pole diagrams used in our complex pole analysis as the so-called “pseudo-pole” diagrams for the transfer function. This methodology can be applied to Prony fits for any system with Prony fits for the input and output. Representative transfer function “pseudo-pole” diagrams are shown in Fig. 6B and C for our system. As demonstrated in Figs. 4 and 5, the pseudo-pole encode information relevant to changes with contractility in hemorrhagic shock, further suggesting that the poles provide information on load independent indices of cardiac function.

We finally aimed to understand whether Bode plots of the numerically determined transfer function from the Prony fits are different at baseline, during hypovolemic shock, and after volume resuscitation. Bode plots were determined by taking the magnitude and phase of the transfer function in the form

(23) $$\bar{Z}(j\omega )=\frac{\left(\sum_{i}C_{i}^{\left(P\right)}\prod_{k\neq i}j\omega \;-\;\mu_{k}^{\left(P\right)}\right)\left(\prod_{i}j\omega \;-\;\mu_{i}^{\left(V\right)}\right)}{\left(\sum_{i}C_{i}^{\left(V\right)}\prod_{k\neq i}j\omega \;-\;\mu_{k}^{\left(V\right)}\right)\left(\prod_{i}j\omega \;-\;\mu_{i}^{\left(P\right)}\right)}$$

Composite transfer functions were averaged for all animals at all time points and Bode plots were plotted between 1 and 100 Hz, which includes physiologically relevant frequencies for rodents. Results are summarized in Fig. 6D and E. Briefly, we observe that the amplitude of the transfer function decays the most with increasing frequency after shock and recovers close to the baseline amplitude after resuscitation. A similar phenomenon is observed for the phase of the transfer function.

## 4. Discussion

This study developed the mathematical foundations for complex pole analysis in the Prony domain of cyclic pressure-volume measurements from the left ventricle of animals subjected to hemorrhage resuscitation with stored RBCs. Primarily, we implemented: (i) a novel convergence algorithm for the Prony fit, thereby allowing us to fit all pressure and volume waveforms with high accuracy (R^2^ > 0.99 in all cases), (ii) a novel pipeline for complex pole analysis in the Prony domain using cyclical biologic and cardiac signals, (iii) novel metrics for the aforementioned complex pole analysis, which demonstrated significant differences after hemorrhagic shock and volume resuscitation, and (iv) a cardiac transfer function using the Prony fits for the pressure and volume signals. The analysis presented here can be leveraged as a common pipeline of Prony analysis for complex biologic and cardiac signals and systems, including pole analysis and transfer function quantification. This pipeline of analysis using the Prony fit may be used to better analyze biologic and medical signals that are collected both in basic science laboratories and clinical settings.

While several studies have utilized the Prony method [2–5,26], to our knowledge, only one recent study focused on mathematical methods for its implementation in MATLAB [3]. Three methods of implementation – least squares [26], total least squares [27], and the matrix pencil method [11] were applied to multifocal visual-evoked potential signals for the diagnosis of multiple sclerosis [3]. Our study only implemented the matrix pencil method, which obtains the poles by finding the eigenvalues of a matrix pencil. We justified utilizing only this implementation as it resulted in the best fit compared to other methods as demonstrated previously [3]. One problem with the Prony fit, however, is that certain aspects of the algorithm are ill-conditioned with significant susceptibility to round-off error [3,17]. We solved this limitation using a gradient ascent-based optimization algorithm, which finds the optimum time decimation factor by maximizing the coefficient of determination of R^2^ between the fit and raw signal. Our algorithm is robust for several signals, as demonstrated by our results but fails to converge for long time courses (>10 s) sampled at rates greater than 1000 Hz.

We provide a detailed analysis for the specific case of femoral artery catheterization pressure waveforms with a comparison to Fourier transform based methods (Fig. 2). Our analysis reveals that the Prony method opts to fit signals by combining high frequency elements out a phase with each other and with dampening. As a result, the provided frequency spectrum is not biologically interpretable. As such, despite the heart rate being 6–7 Hz in rodents, the Prony frequency spectra does not pass 4 Hz. Moreover, the bandwidth predicted by the Prony signal is not Bandwidth in the traditional sense. Nyquist theory for the Prony fit does not restrict the range or spacing of frequencies, as demonstrated by the approximate 500 Hz bandwidth in the pole diagram, but rather the total number of dampened sinusoids that can be fit. Specifically, any range and combinations of dampened sinusoids of any frequencies and bandwidth can be used, so long as the total number of dampened sinusoids is less than a quarter of the sampling frequency. This, however, does have the added benefit of eliminating edge effects compared to the Fourier series decomposition. We have summarized our findings comparing the two algorithms in Table 1. Using our implementation of the Prony method, we can replicate a Fourier series decomposition by evenly sampling poles on the unit circle. Those poles in the real-imaginary plane on the unit circle correspond to zero dampening, which result in sine waves. This finding further highlights the intimate relationship between the two transforms.

Our study is the first to apply the Prony method to cyclic pressure-volume waveforms from catheterization data, though several studies have implemented the algorithm. In one previous study, Chen used the total least squares implementation of the Prony method for discrimination of ventricular fibrillation (VF), ventricular tachycardia (VT), and supraventricular tachycardia (SVT) [4]. While modern machine learning methods are superior for this task [21,28–31], this study still boasted an accuracy of 95%, 96%, and 98% for SVT, VT, and VF, respectively. Importantly, this study highlights that for cyclic cardiac signals, the Prony method provides a useful tool for signal processing. Future studies should further investigate and expand upon the role of Prony analysis for analysis of pressure-volume measurements. In addition, future studies that also combine the Prony method with modern machine learning techniques may prove to be useful for such analyses.

In our pressure-volume analysis, our results demonstrate that differences in the poles of the pressure and volume waveforms can be used to distinguish between hypovolemic shock and volume resuscitation. We hypothesize that because hypovolemic shock results in minor changes in cardiac mechanics, namely increased contractility, decreased systemic vascular resistance, decreases in filling pressure, and decreased end systolic [8]. Such a relationship requires validation in future studies. In addition, future studies should aim to investigate application of Prony based methods to pressure-volume measurements to hereditary cardiomyopathies (e.g., hypertrophic cardiomyopathy and dilated cardiomyopathy) and structural heart disease, which may yield more drastic changes to Prony pole diagrams.

### 4.1. Limitations

Our study has several limitations. The Prony method has several numerical limitations, which we attempted to solve with a gradient ascent-based optimization algorithm. All our analyses were performed on signals of 1 s in length, and we are unable to quantify the individual dispersion processing larger signals given limitations with recordings of the signal during animal experimentation. Nonetheless, our implementation of the matrix pencil method for the Prony algorithm was stable in all conditions we tested. We were not powered to identify certain changes in the pole diagrams during hypovolemic shock and after volume resuscitation, which may yield Type II error. While we were able to identify few differences, future studies should apply the method to more appropriate conditions for larger differences. Importantly, our study was validated for rat hemodynamic data. For use in human hemodynamic data, recordings may be roughly six times longer (< 60 s) given the difference in heart rate between humans (60 bpm) and rats (360 bpm).

## 5. Conclusion

This paper introduced a pipeline to analyze biologic signals with Prony analysis. Unique to this pipeline is the following: (i) We implanted a gradient ascent-based optimization algorithm, which optimizes the R^2^ between the fit and raw signal, which allows for efficient convergence of the Prony algorithm. (ii) We developed novel methods for Prony pole analysis including algorithms to find the location of the highest concentration of optimal poles, their dispersion, and the number of poles and distance from the unit circle, all of which served to reveal differences between shock and resuscitation. (iii) We derived a cardiac transfer function using pressure and volume signals in the Prony domain, which were found to be distinct at baseline, shock, and resuscitation. Future directions should aim to apply this pipeline in cardiac pathophysiologies which change the structure of the heart such as hereditary cardiomyopathies and structural heart disease.

## Supplementary Material

supplemental

Supplementary material associated with this article can be found, in the online version, at doi:10.1016/j.medengphy.2023.103987.

## Figures and Tables

**Table 1:** Comparison of the Prony series decomposition and the Fourier series decomposition.

	Prony series decomposition	Fourier series decomposition

STRENGTHS	- High fidelity fit - No edge effects - Able to fit low resolution signals - Returns complex poles - Generates analytical functions for signals	- Few numerical limitations - Provides interpretable frequency spectra - Returns sine or cosine series suitable for simple analyses
WEAKNESSES	- Frequency spectrum is not interpretable - Susceptible to numerical errors	- Edge effects are prominent - Requires highly sampled signals - Does not provide system poles
