# Supplementary material for: Prony Analysis of Left Ventricle Pressure and Volume
Source: Med Eng Phys. Author manuscript; Available in PMC 2026 Feb 16. (PMC12908439; doi:10.1016/j.medengphy.2023.103987)
Supplement: supplemental [file NIHMS2138960-supplement-supplemental.docx]

**
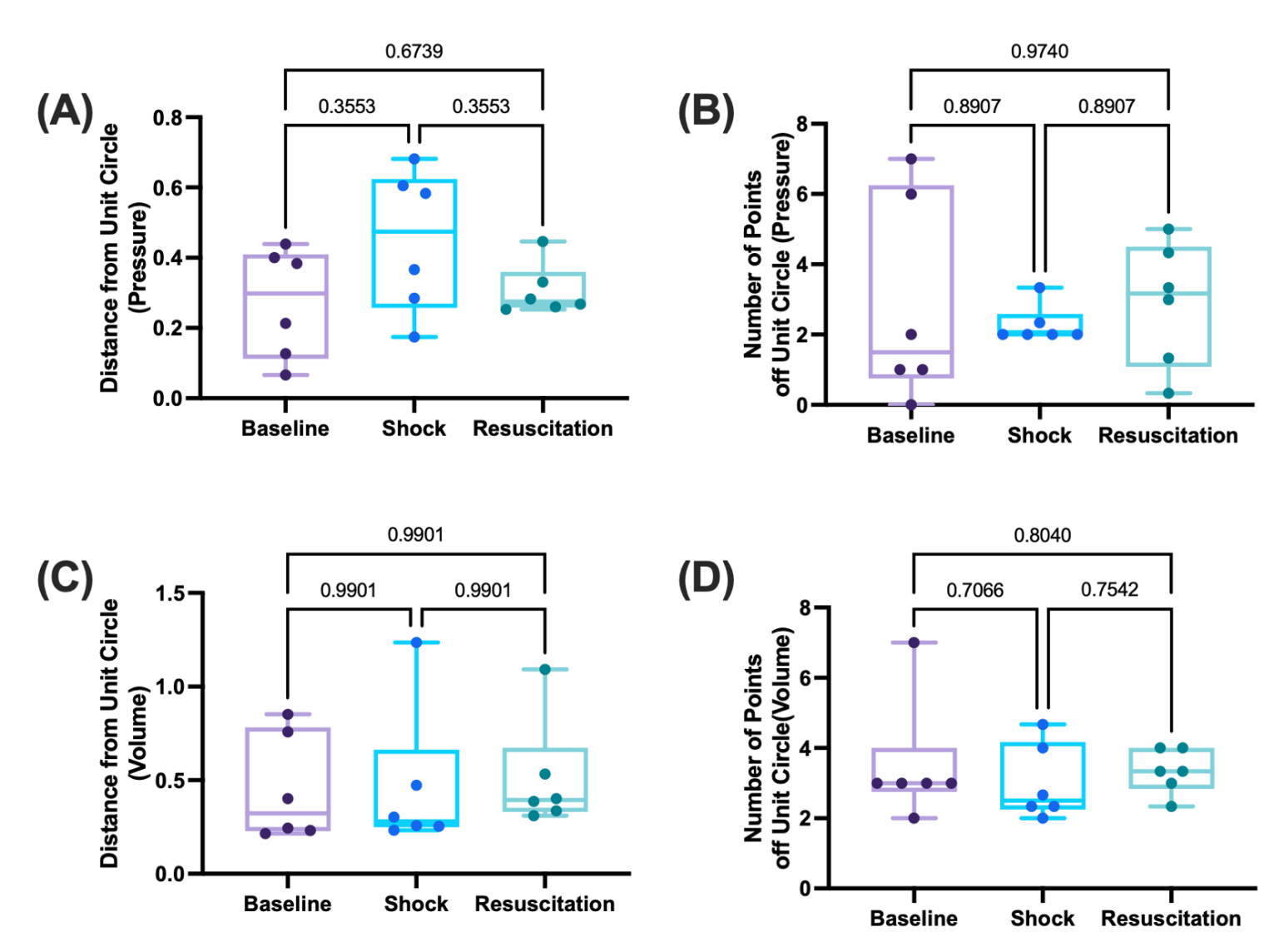
**

**Supplemental Figure 1. Complex Pole Analysis at Baseline, Shock, and Resuscitation for only stable Prony poles.** Summary data for the (A) mean distance for the top five further poles and (B) number of poles off the unit circle for the pressure waveforms, and (C) mean distance for the top five further poles and (D) number of poles off the unit circle volume waveform are shown. Results from this analysis were compared using one-way repeated measures ANOVA and mixed effect models with Holm-Sidak’s test for post-hoc comparison when appropriate.


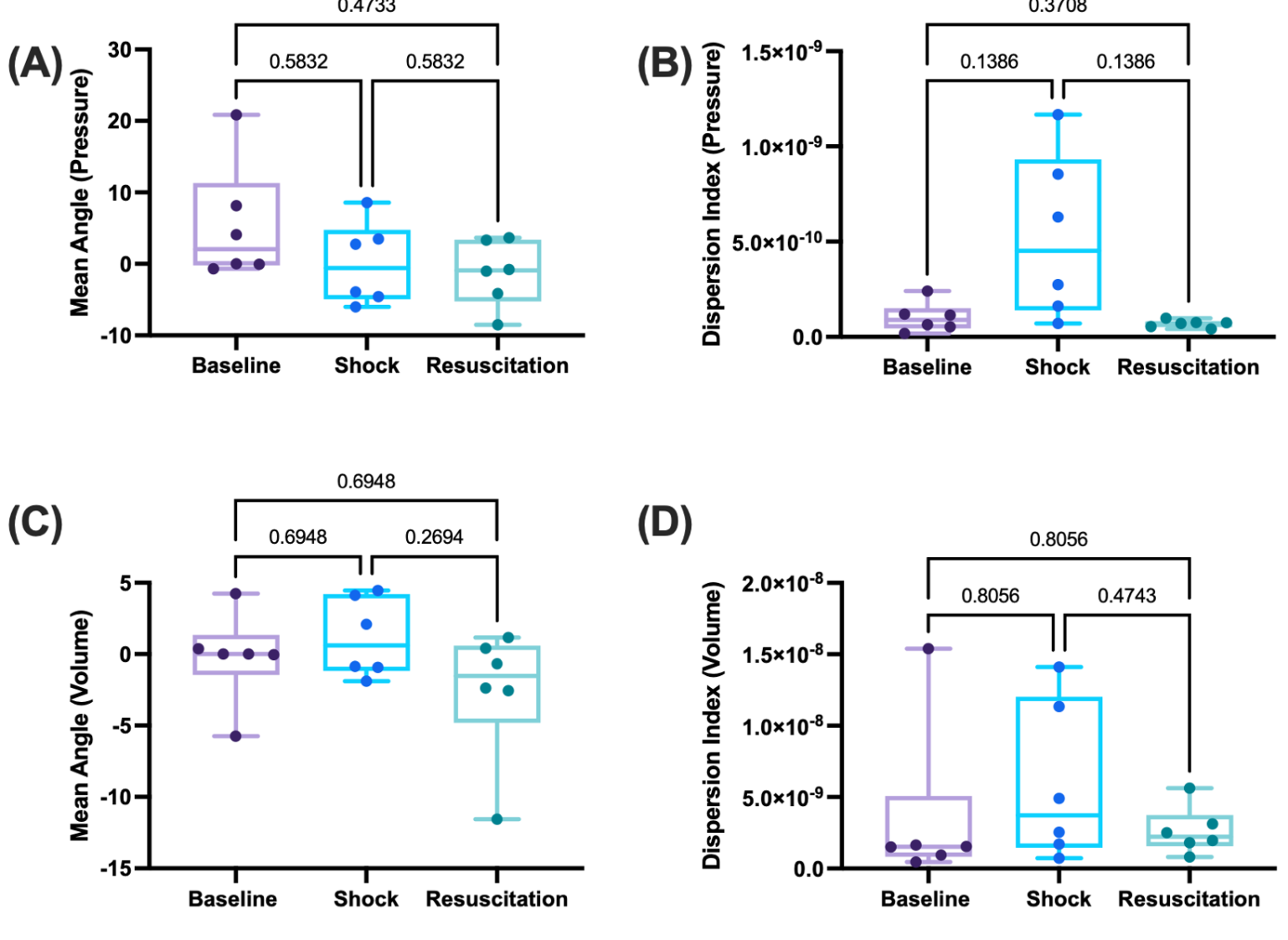


**Supplemental Figure 2. Complex Pole Analysis at Baseline, Shock, and Resuscitation for both stable and unstable Prony poles.** Summary data for the (A) mean angle and (B) dispersion for the pressure waveforms, and (C) mean angle and (D) dispersions for the volume waveform are shown. Results from this analysis were compared using one-way repeated measures ANOVA and mixed effect models with Holm-Sidak’s test for post-hoc comparison when appropriate.


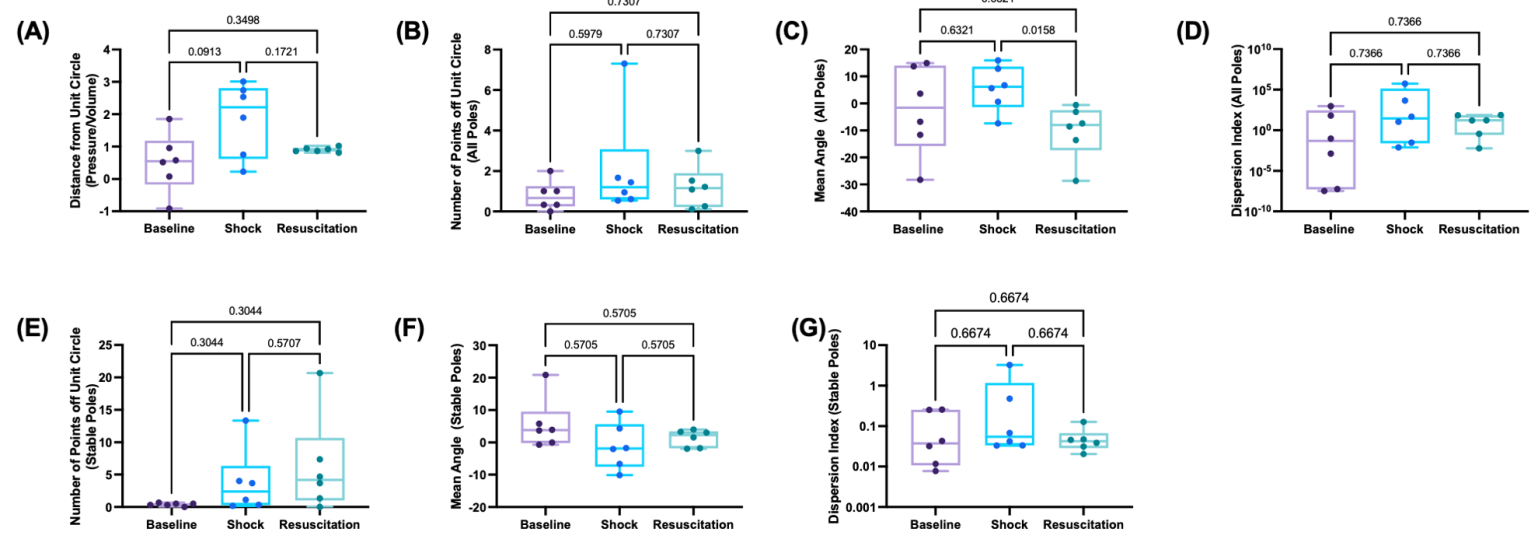


**Supplemental Figure 3**. **Complex Pole Analysis at Baseline, Shock, and Resuscitation for both stable and unstable poles**. For each metric described, the ratio (or difference in the case of the mean angle metric) was determined between the pressure and volume waveform. (A) Distance from the unit circle, (B) number of poles of the unit circle, (C) mean angle, and (D) dispersion index were assessed for all Prony poles. Results from this analysis were compared using one-way repeated measures ANOVA and mixed effect models with Holm-Sidak’s test for post-hoc comparison when appropriate. Similarly, (E) the number of poles off the unit circle, (F) the mean angle, and (G) the dispersion were assessed for only stable Prony poles. Results from this analysis were compared using one-way repeated measures ANOVA and mixed effect models with Holm-Sidak’s test for post-hoc comparison when appropriate.
